# Subchronic Arsenite Exposure Induced Atrophy and Erythropoietin Sensitivity Reduction in Skeletal Muscle Were Relevant to Declined Serum Melatonin Levels in Middle-Aged Rats

**DOI:** 10.3390/toxics11080689

**Published:** 2023-08-10

**Authors:** Xiong Chen, Wanying Chen, Dapeng Wang, Lu Ma, Junyan Tao, Aihua Zhang

**Affiliations:** The Key Laboratory of Environmental Pollution Monitoring and Disease Control, Ministry of Education, School of Public Health, Guizhou Medical University, Guiyang 550025, China; cwy2676@163.com (W.C.);

**Keywords:** arsenic, skeletal muscle, myofiber, melatonin, EPO sensitivity

## Abstract

Arsenic is a kind of widespread environmental toxicant with multiorgan-toxic effects, and arsenic exposure is associated with the occurrence and development of many chronic diseases. The influence of environmental arsenic exposure on skeletal muscle, which is a vital organ of energy and glucose metabolism, has received increasing attention. This study aimed to investigate the types of inorganic arsenic-induced skeletal muscle injury, and the potential regulatory effects of melatonin (MT) and erythropoietin (EPO) in young (3-month-old) and middle-aged (12-month-old) rats. Our results showed that 1 mg/L sodium arsenite exposure for 3 months could accelerate gastrocnemius muscle atrophy and promote the switch of type II fibers to type I fibers in middle-aged rats; however, it did not cause significant pathological changes of gastrocnemius muscle in young rats. In addition, arsenite could inhibit serum MT levels, and promote serum EPO levels but inhibit EPO receptor (EPOR) expression in gastrocnemius muscle in middle-aged rats, while serum MT levels and EPOR expression in gastrocnemius muscle showed an opposite effect in young rats. Importantly, exogenous MT antagonized the arsenite-induced skeletal muscle toxic effect and restored serum EPO and gastrocnemius muscle EPOR expression levels in middle-aged rats. There was a positive correlation among gastrocnemius muscle index, serum MT level, and gastrocnemius muscle EPOR protein level in arsenite-exposed rats. This study demonstrated that inorganic arsenic could accelerate skeletal muscle mass loss and type II fiber reduction in middle-aged rats, which may be related to decreased MT secretion and declined EPO sensitivity in skeletal muscle.

## 1. Introduction

Arsenic is one of the most potent hazardous substances that may enter the food chain through arsenic-contaminated groundwater or naturally arsenic-riched soils, threatening the health of approximately 200 million people around the world [1,2]. Long-term excessive exposure to environmental arsenic may exert multiple toxic effects on living organisms, and even a variety of chronic diseases [3,4,5]. Recent epidemiological studies have shown that arsenic exposure may be associated with loss of skeletal muscle mass among individuals from arsenic-endemic areas of Bangladesh [6,7]. In vivo and in vitro studies have demonstrated that arsenic trioxide treatment significantly decreased the muscle mass of mice via drinking water for 4 weeks, and inorganic arsenic inhibited myogenic differentiation and muscle regeneration and induced myotube atrophy [8,9,10]. However, the pathological types and underlying mechanisms of inorganic arsenic-induced skeletal muscle injury remain largely unknown.

The skeletal muscle is the largest organ in the body and plays an important role in health and longevity, for even small changes can have a tremendous impact on whole-body metabolism [11,12]. Skeletal muscle is characterized by age-related decline in mass, which begins to decline around age 40 to 50 and increases the risk of chronic diseases in elder adults, including chronic respiratory disease, cardiovascular disease, and diabetes [13]. Skeletal muscle is heterogeneous tissue and comprises different types of muscle fibers. According to their contractile properties, mammalian skeletal muscle fibers can be divided into slow-twitch muscle fibers (type I) and fast-twitch muscle fibers (type II) [14]. Under the influence of environmental stress, nutritional deficiency, and an unhealthy lifestyle, the two types of skeletal muscle fiber can switch from each other [15]. However, the influence of arsenic exposure on skeletal muscle fiber types and type switching is unclear.

Erythropoietin (EPO) is a nephrogenic hormone that acts as a major regulator of erythropoiesis and also a critical oxygen-regulated hormone in response to physical or metabolic stress [16]. Physiologically, EPO secreted by the kidney plays a key role in the maturation of red blood cells [17]. It has recently been reported that EPO has regulatory effects on both myocardium and skeletal muscle and has several beneficial effects on the repairment of skeletal muscle injury and prevention of fibrosis, with increased genes related to muscle hypoxia and cell death, while decreased genes related to glycolysis and mitochondrial function [18,19,20]. EPO primarily exerts its effect by binding EPO receptor (EPOR), which is a cytokine class I receptor superfamily member. EPORs are expressed in a variety of tissues, including erythrocyte progenitor, skeletal muscle, neurons, and other tissues, which is related to the sensitivity of EPO [21]. Therefore, in addition to hematopoietic tissue, EPO also plays an active role in a variety of non-hematopoietic tissues through interaction with the EPOR, especially skeletal muscle [22]. Although recent studies have shown that inorganic arsenic can promote the secretion of EPO [23,24], more study is needed to determine whether arsenic exposure can affect the sensitivity to EPO in multiple organs, such as skeletal muscle.

Melatonin (MT) is an endogenous amine hormone primarily secreted by the pineal gland. The physiological function of MT is to maintain sleep quality and endocrine and immune system homeostasis [25]. Many studies have shown that MT may serve as a potential agent in the treatment of sarcopenia, as it exerts a skeletal muscle protection effect through multiple pathways [26,27]. Importantly, studies have shown that MT can promote EPO sensitivity in anemic patients with chronic kidney disease or chronic renal failure [28,29]. However, it remains to be determined whether arsenic can affect endogenous MT secretion and whether exogenous MT can antagonize arsenite-induced skeletal muscle toxicity.

In the present study, we investigated the effect of environmental concentration arsenic exposure on endogenous MT secretion and the correlations among MT secretion level, EPO sensitivity of skeletal muscle, and gastrocnemius muscle index in rats.

## 2. Materials and Methods

### 2.1. Animals

Forty male Specified Pathogen Free (SPF)-grade Sprague Dawley (SD) rats (including sixteen 3-month-old rats and twenty-four 12-month-old rats) were purchased from Experimental Animal Centre of Guizhou Medical University. All rats were fed adaptively in communal plastic cages for one week before the experiment began at Guizhou Medical University Experimental Animal Centre. The cages were separately placed in a climate-controlled room (21 ± 2 °C), maintained on a 12 h/12 h light/dark cycle, and given food and water ad libitum. All experimental protocols were performed in accordance with and approved by the Animal Care Welfare Committee of Guizhou Medical University, Guiyang, China, No. 2100398.

The dose of arsenite (1 mg/L) was selected based on a previous study that arsenic concentration in groundwater of some countries reached a high level at 9900 µg/L (9.9 mg/L) or even 100,000 µg/L (100 mg/L) [30]. Moreover, the arsenite exposure duration was also chosen based on previous studies [31,32,33]. In the present study, therefore, we used a low level of environmental arsenic concentration (1 mg/L) for subchronic exposure in rats. Sodium arsenite was purchased from Sigma (St. Louis, MO, USA, purity: 99.0%).

### 2.2. Experimental Design of the Animal Study

According to different months of age and body weight, the rats were initially randomly divided into 4 groups: young normal control group (initially from 3-month-old), young arsenite-exposed group (initially from 3-month-old), middle-aged normal control group (initially from 12-month-old) and middle-aged arsenite-exposed group (initially from 12-month-old), with 8 rats in the first three groups and 16 rats in the fourth group. Control groups were exposed to drinking deionized water freely for 3 months. The rats in the young arsenite-exposed group were exposed to 1 mg/L arsenite by free drinking water for 3 months, while the rats in the middle-aged arsenite-exposed group were exposed to 1 mg/L arsenite for 2 months and then they were randomly divided into 2 groups (*n* = 8 rats per group), namely middle-aged arsenite-exposed group and middle-aged MT intervention group respectively. The former was intraperitoneal injection with normal saline for 1 month (every day), while the latter was given 10 mg/kg MT by intraperitoneal injection for 1 month (every day) while being exposed to arsenite [34,35]. The body weights (g) of the rats in each group were recorded weekly.

At the end of the experiment, individual rats were placed in metabolic cages for urine collection and then rats were anesthetized with 3% pentobarbital sodium. Blood samples were collected from the heart for the preparation of serum (2 h of clotting followed by 10 min centrifuged at 3000 g). Serum was carefully removed and transferred to clean 1.5 mL Eppendorf Tubes for storage at −80 °C. Gastrocnemius muscle of both legs was carefully harvested and weighed. After weighing the gastrocnemius muscle tissues, the left tissues were divided into two parts and fixed with paraformaldehyde overnight or frozen at −80 °C. Among them, the tissues fixed by paraformaldehyde were used for HE staining, Masson staining, and Immunofluorescence Assays, while the tissues frozen at −80 °C were used for Oil red O staining and the determination of arsenic content in tissues. In addition, the right tissues were divided into two parts, which were used for protein extraction and Genomic DNA Isolation, respectively. ELISA assay was used to detect related indexes in serum. The gastrocnemius muscle index (gastrocnemius muscle index = gastrocnemius muscle weight/body weight × 100%) was calculated.

### 2.3. Determination of Total Arsenic

The concentrations of arsenic in the urine and gastrocnemius muscle were analyzed by inductively coupled plasma mass spectrometry (ICP-MS) using a PerkinElmer/Sciex ELAM ICPMS (PerkinElmer, Valencia, CA, USA). For the pretreatment of urine samples, the urine was filtered with a 0.2 μm inorganic filter, diluted with 2% HNO_3_, and put on the machine. For pretreatment of muscle tissue samples, 0.100 g (accurate to 0.001 g) sample was weighed and placed in the microwave digestion tank, 5 mL HNO_3_ and 2 mL H_2_O_2_ were added and placed for 2 h, and then placed in the microwave digestion instrument for digestion. Step 1: heating time 15 min, holding temperature 140 °C, holding time 2 min. Step 2: heating time 10 min, holding time 190 °C for 30 min. After cooling to room temperature, the samples were removed, and the acid was removed to about 2 mL at 140 °C. Then the samples were transferred and fixed to 10 mL with 2% HNO_3_. Appropriate protocol blanks and standards for arsenic were used to ensure internal quality control and assurance of the analysis. Further, the operating conditions were checked at regular intervals using calibration standards and laboratory blanks. The arsenic concentrations were expressed as μg per l in urine (as the sum of arsenic metabolites, U-As) and μg per g tissue wet weight in gastrocnemius muscle (GM-As).

### 2.4. Enzyme-Linked Immunosorbent Assay (ELISA) Assays

Serum levels of EPO (ER0170) were measured using enzyme-linked immunosorbent assays (ELISA) plates (Fine Biotech Co., Ltd., Wuhan, China). The sensitivity and intraassay coefficient of variation of EPO was 37.5 pg/mL and 8.0% respectively. Serum levels of MT (E-EL-R0031c) were assayed using ELISA plates (Elabscience Biotechnology Company, Wuhan, China) according to the manufacturer’s handbook. The sensitivity and intraassay coefficient of variation of MT was 18.75 pg/mL and 4.5% respectively. The absorbance from each sample was measured in duplicate using a spectrophotometric microplate reader at a wavelength of 450 nm (Thermo Fisher Scientific, Shanghai, China). Serum samples were tested in duplicate within one assay, and the results were expressed in terms of rats’ MT standard (ng/L) and EPO standard (ng/L).

### 2.5. Histology Assessment

The gastrocnemius muscle tissue of the SD rats was dissected rapidly and in a 4% formaldehyde solution for histological analyses. After fixation, muscle tissues were cleaned and immersed in 4% fresh paraformaldehyde for an additional 24 h following cut muscle tissues into 2–3 mm thickness and embedded in paraffin. Then, muscle tissues were dehydrated conventionally, dewaxed with a xylene substitute, and hydrated. After that, paraffin-embedded muscle tissues were cut into 4 μm sections using a slicer (Leica, Wetzlar, Germany). Subsequently, sections were subjected to hematoxylin and eosin (H&E), Masson’s trichrome staining as per the standard laboratory protocols.

The gastrocnemius muscle tissue was post-fixed overnight at 4 °C and then immersed in 30% sucrose solution in PBS for cryoprotection. Serial 25 µm thick coronal sections were cut on a freezing sliding microtome (Thermo, Shanghai, China), and stored in a cryoprotectant (25% ethylene glycol, 25% glycerol, and 0.05 M phosphate buffer) at 4 °C until use. An oil red O kit (Servicebio, Wuhan, China) was used to detect lipid droplet accumulation in skeletal muscle. According to the instructions of the kit, the lipid droplets were dyed red, and the nuclei were stained dark blue. The morphological characteristics of skeletal muscle fibers were assessed using an optical microscope (EX20, SUNNY, Ningbo, China) by examining five randomly selected fields (×200).

### 2.6. Immunofluorescence Assay

To investigate the effect of arsenite exposure on skeletal muscle fiber type switching (type I and type II), immunofluorescence was performed in one paraffin section for two markers. Sections were dewaxed, rehydrated, and immersed in the slides in EDTA antigen retrieval buffer (pH 8.0) and maintained at a sub-boiling temperature for 8 min, standing for 8 min, and then followed by another sub-boiling temperature for 7 min. We ensured that the buffer solution did not evaporate. After air was cooling, the sections were washed three times with PBS (pH 7.4) and then immersed in 3% H_2_O_2_ and incubated at room temperature for 25 min in a dark place. They were washed again and obvious liquid was eliminated, followed by covering the objective tissues with 3% BSA to throw away the blocking solution slightly. All samples were subjected to overnight incubation (12–15 h at 4 °C) with Anti-Fast Myosin Skeletal Heavy chain Rabbit pAb (1:2000, diluted with PBS appropriately). All sections were then incubated with HRP conjugated Goat Anti-Rabbit IgG (H + L) at room temperature for 50 min in dark conditions. Next, we incubated the slides with a TSA-CY3 solution for 10 min in dark conditions. After that, slides were washed with TBST. These slides were microwaved again to remove the primary antibodies and secondary antibodies combined with tissues. Afterward, slides were incubated with Anti-Slow Skeletal Myosin Heavy chain Rabbit pAb (1:200, diluted with PBS appropriately) overnight at 4 °C, covered the objective tissue with Alexa Fluor^®^ 488-conjugated Goat Anti-Rabbit IgG (H + L). Finally, nuclei were stained using DAPI, and slides were coverslipped with an anti-fade mounting medium. Microscopy detection and image collection were performed via Fluorescent Microscopy. DAPI glows blue after UV excitation at a wavelength of 330–380 nm and an emission wavelength of 420 nm. FITC glows green after excitation at a wavelength of 465–495 nm and an emission wavelength of 515–555 nm. CY3 glows red after excitation at a wavelength of 510–560 nm and an emission wavelength of 590 nm. The morphological characteristics of gastrocnemius muscle fibers were observed at 200 magnification, and then the quantification of myofiber cross-sectional areas and muscle fiber numbers was analyzed by Image J.

### 2.7. Genomic DNA Isolation and Telomere Length Analysis

TIANamp Genomic DNA kit (Tiangen, Beijing, China) was used to extract genomic DNA from gastrocnemius muscle. DNA concentration was measured using a microplate reader. Samples were quantified to a final concentration of 25 ng/1.5 µL to measure telomere length. qPCR was performed using SuperReal PreMix (SYBR Green, Tiangen, Beijing, China). Primers used were as follows: forward TEL 5′-GGT TTT TGA GGG TGA GGG TGA GGG TGA GGG TGA GGG t-3′, reverse TEL 50-TCC CGA CTA TCC CTA TCC CTA TCC CTA TCC CTA TCC CTA-30, forward AT1 5′-ACG TGT TCT CAG CAT CGA CCG CTA CC-3′, and reverse AT1 5′-AGA ATG ATA AGG AAA GGG AAC AAG AAG CCC-3′. The relative telomere length was measured by comparing the ratio of telomere repeat copy number (T as Tel1) and single-gene copy number (S as AT1), expressed as telomere length (T/S) ratio. Each value obtained by qPCR was processed through the formula T/S = 2^−∆Ct^, where ∆CT = CT_telomere_ − CT_AT1_. Each ratio was then compared with the ratio of the reference DNA. Each DNA sample collected was measured in duplicate.

### 2.8. Western Blot

Total protein was extracted from muscle tissues using RIPA buffer (Beyotime Technology, Shanghai, China) containing 1 mM PMSF (Beyotime Technology, Shanghai, China) and quantified by the BCA Protein Assay Kit (Beyotime, Shanghai, China). The extracts were added to the loading buffer (Beyotime Technology, Shanghai, China) and denatured by boiling at 100 °C for 10 min. An equal amount of 20 μg protein was loaded onto a 10% SDS-PAGE gel (Epizyme Biotech, Shanghai, China) for electrophoresis and transferred to a PVDF membrane (Millipore, Bedford, MA, USA). The membranes were routinely washed and blocked with 5% non-fat milk, oscillating at room temperature for 2 h, and incubated overnight with antibodies against Anti-Atrogin-1 antibody (1:1000, ab168372, Abcam, Cambridge, UK), Anti-EPOR antibody (1:1000, E-AB-123926, Elabscience, Wuhan, China) and β-Actin (1:2000, 66009-1-ig, ProteinTech, Wuhan, China) at 4 °C. After rinsing in TBST, the membranes were incubated with secondary antibodies (1:10000, SA00001-1, SA00001-2, ProteinTech, Wuhan, China) and oscillated at room temperature for 2 h. β-Actin levels were measured as an internal control. A Bio-Rad imaging system and Image J software were used to detect the immunoreactive bands and to quantify each sample.

### 2.9. Statistical Analysis

All data are expressed as the mean ± standard deviation (SD). Two-way analysis of variance (ANOVA) and one-way analysis of variance (ANOVA) between groups were used to evaluate the difference between groups, and the correlation statistics were performed by the Pearson’s correlation analysis. A *p*-value less than 0.05 was considered to indicate statistical significance. GraphPad Prism 6.0 (La Jolla, CA, USA) was used for the statistical analyses.

## 3. Results

### 3.1. Subchronic Arsenite Exposure Reduced Skeletal Muscle Index in Middle-Aged Rats

**Figure 1:**
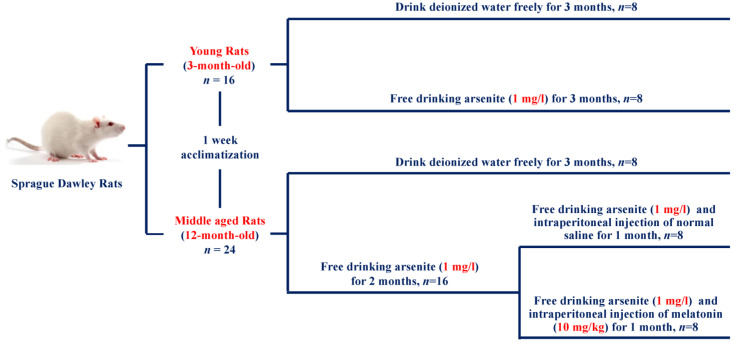
Schematic illustration of the study design. SD rats were randomly divided into four groups: young normal control group (initially from 3-month-old), young arsenite-exposed group (initially from 3-month-old), middle-aged normal control group (initially from 12-month-old), and middle-aged arsenite-exposed group (initially from 12-month-old). Control groups were exposed to drinking deionized water freely for 3 months. The rats in the young arsenite-exposed group were exposed to 1 mg/L arsenite for 3 months, while the rats in the middle-aged arsenite-exposed group were exposed to 1 mg/L arsenite for 2 months and then were randomly divided into two groups, namely middle-aged arsenite-exposed group and middle-aged MT intervention group, respectively. The former group was intraperitoneally injected with normal saline for 1 month, while the latter was given 10 mg/kg MT for 1 month while being exposed to arsenite.

To illuminate the effect of subchronic arsenite exposure on skeletal muscle, the 3-month-old rats and 12-month-old rats were exposed to 1 mg/L arsenite for 3 months, the pathological changes in gastrocnemius muscle were observed, and related protein changes in peripheral blood and gastrocnemius muscle tissue were detected. In addition, the 12-month-old rats were treated with 10 mg/kg MT by intraperitoneal injection for one month after 2 months of arsenite exposure. The experimental design is described in Figure 1.

**Figure 2:**
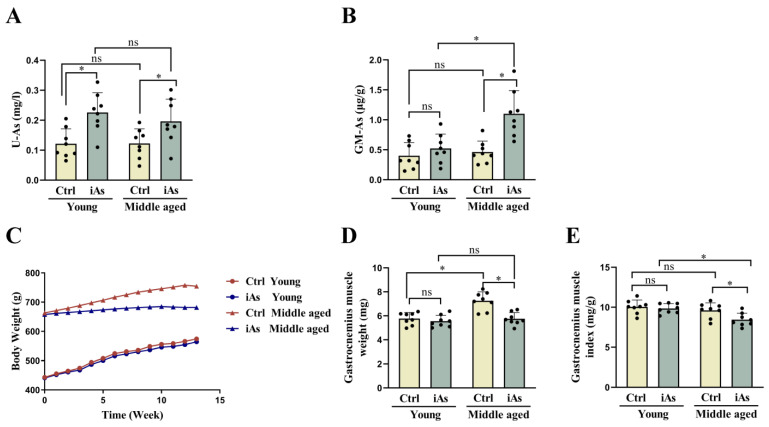
Impact of 1 mg/L arsenite exposure on arsenic concentrations in urine (**A**), gastrocnemius muscle (**B**), body weight (**C**), gastrocnemius muscle weight (**D**), and gastrocnemius muscle index (**E**) in 3- and 12-month-old rats. Ctrl, Control; iAs, Arsenite; U-As, Urine arsenic concentration; GM-As, Gastrocnemius muscle arsenic concentration. The data are expressed as mean ±SD for eight rats per group. ns, no significance compared to the control group. * *p* < 0.05, significant difference compared to the control group.

Firstly, the concentrations of urine and gastrocnemius muscle arsenic were detected to figure out the extent of arsenic accumulation in rats. Compared with their respective control group, arsenite exposure for 3-month-old rats induced an increase in arsenic concentration in urine but induced no significant change in gastrocnemius muscle, whereas 12-month-old rats had increased arsenic concentrations both in urine and gastrocnemius muscle (Figure 2A,B). The results suggested an accumulation of arsenic in the skeletal muscle of 12-month-old rats.

Secondly, the body and gastrocnemius muscle weights were measured to evaluate the influence of arsenite on skeletal muscle mass in rats. Compared with their respective control group, the body weight of 12-month-old arsenite-exposed rats decreased significantly, while there was no statistical change in 3-month-old arsenite-exposed rats (Figure 2C). The gastrocnemius muscle weight and index decreased significantly in 12-month-old arsenite-exposed rats but had no significant change in 3-month-old arsenite-exposed rats (Figure 2D,E).

### 3.2. Arsenite Exposure Accelerated Skeletal Muscle Atrophy and Telomere Shortening in Middle-Aged Rats

**Figure 3:**
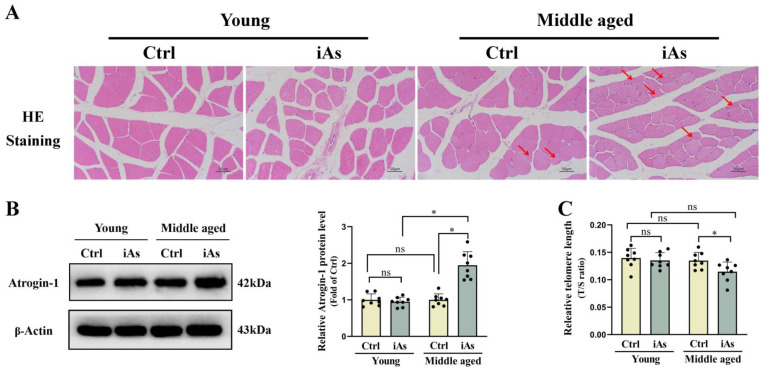
Impact of 1 mg/L arsenite exposure on pathological morphology (**A**), the protein level of Atrogin-1 (**B**), and the relative telomere length (**C**) of the gastrocnemius muscle in 3- and 12-month-old rats. Ctrl, Control; iAs, Arsenite. The data are expressed as mean ±SD for eight rats per group. Images (×200 magnification). Red arrows indicate atrophied skeletal muscle fibers. Scale bars represent 50 µm. ns, no significance compared to the control group. * *p* < 0.05, significant difference compared to the control group.

As shown in the H-E staining results, in the control groups and 3-month-old arsenite-exposure group, there were many myofibrils arrayed parallel along the long axis of the cells, and the nuclei were elliptical near the myofilm. However, in the 12-month-old arsenite-exposure group, the interval of muscle fibers in some of the muscle bundles of muscle widened, a portion of myofibers of gastrocnemius muscle had atrophied, became thinner and longer, the edges increased, the appearance was irregular, and the nuclei increased and arranged in aggregation (Figure 3A). Western blot results showed that the level of muscle atrophy associated protein Atrogin-1 was significantly upregulated in 12-month-old arsenite-exposed rats, but had no change in 3-month-old arsenite-exposed rats (Figure 3B). We also measured the relative telomere length of gastrocnemius muscle to reflect the influence of arsenic exposure on skeletal muscle degradation. The results showed that the relative telomere length was shortened in 12-month-old arsenite-exposed rats, but had no change in 3-month-old arsenite-exposed rats (Figure 3C).

### 3.3. Arsenite Exposure Induced Type II to Type I Myofiber Switching in Middle-Aged Rats

**Figure 4:**
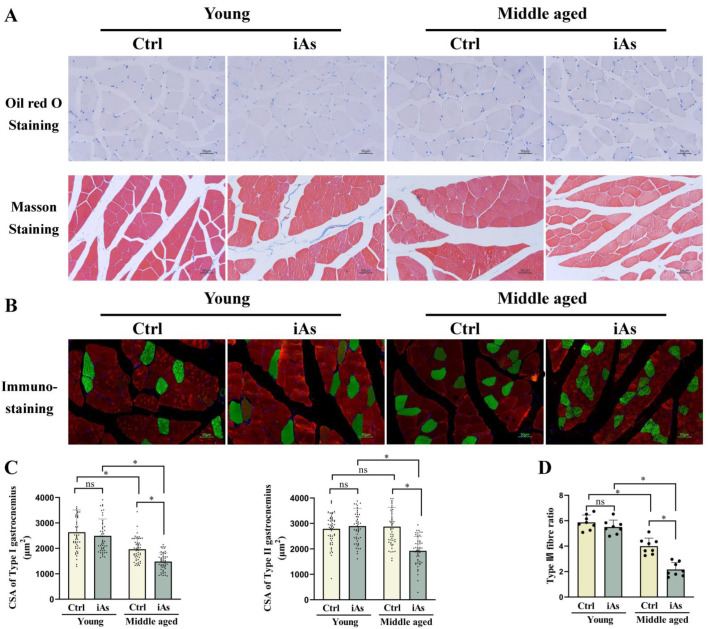
Impact of 1 mg/L arsenite exposure on lipid accumulation and collagen fiber deposition (**A**), gastrocnemius muscle fiber cross-sectional area (**B**,**C**) and myofiber type switching (**B**,**D**) in 3- and 12-month-old rats. Ctrl, Control; iAs, Arsenite. The data are expressed as mean ±SD for eight rats per group. Images (×200 magnification). The green represents type I (slow-twitch) skeletal muscle fiber and the red represents type II (fast-twitch) skeletal muscle fiber. Scale bars represent 50 µm. ns, no significance compared to the control group. * *p* < 0.05, significant difference compared to the control group.

To further clarify the pathological types of arsenite-induced skeletal muscle injury, we performed Oil red O Staining, Masson Staining, and Immunostaining to reflect the lipid accumulation, collagen fiber deposition, and muscle fiber changes in gastrocnemius muscle tissue, respectively. Oil red O Staining and Masson Staining results showed that there was no obvious fatty degeneration and fibrosis observed in the gastrocnemius muscle tissue in either 3- or 12-month-old arsenite-exposed rats (Figure 4A). Immunostaining results further showed that both type I and II gastrocnemius muscle fiber cross-sectional area (CSA) as well as the ratio of type II/I myofiber number were decreased in 12-month-old arsenite-exposed rats, but had no change in 3-month-old arsenite-exposed rats (Figure 4B–D).

### 3.4. Influence of Arsenite Exposure on Serum Levels of MT and EPO in Rats

**Figure 5:**
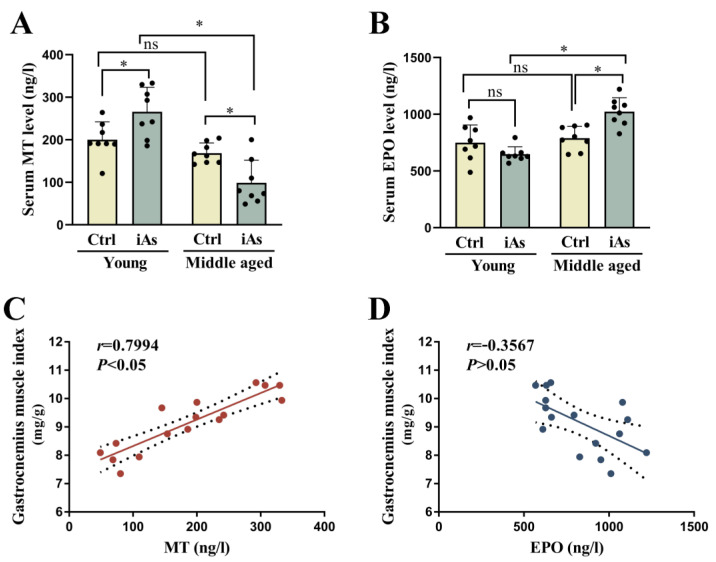
Impact of 1 mg/L arsenite exposure on serum MT and EPO level (**A**,**B**), as well as their correlations with gastrocnemius muscle index (**C**,**D**) in 3- and 12-month-old rats. The gastrocnemius muscle index was positively correlated with serum MT level (*n* = 16, *r* = 0.7994, *p* < 0.05), but did not correlate with serum EPO level (*n* = 16, *r* = −0.3567, *p* > 0.05). Ctrl, Control; iAs, Arsenite; MT, Melatonin; EPO, erythropoietin. The data are expressed as mean ±SD for eight rats per group. ns and *p* > 0.05, no significance compared to the control group. * *p* < 0.05, significant difference compared to the control group.

The serum level of MT was significantly decreased while the serum level of EPO was significantly increased in 12-month-old arsenite-exposed rats, and the serum level of MT was upregulated while the serum level of EPO had no statistically significant difference in 3-month-old arsenite-exposed rats (Figure 5A,B). The correlation analysis showed that serum MT level was positively correlated with gastrocnemius muscle index, while serum EPO level did not correlate with gastrocnemius muscle index (Figure 5C,D).

### 3.5. Influence of Arsenite Exposure on EPOR Expression of Skeletal Muscle in Rats

**Figure 6:**
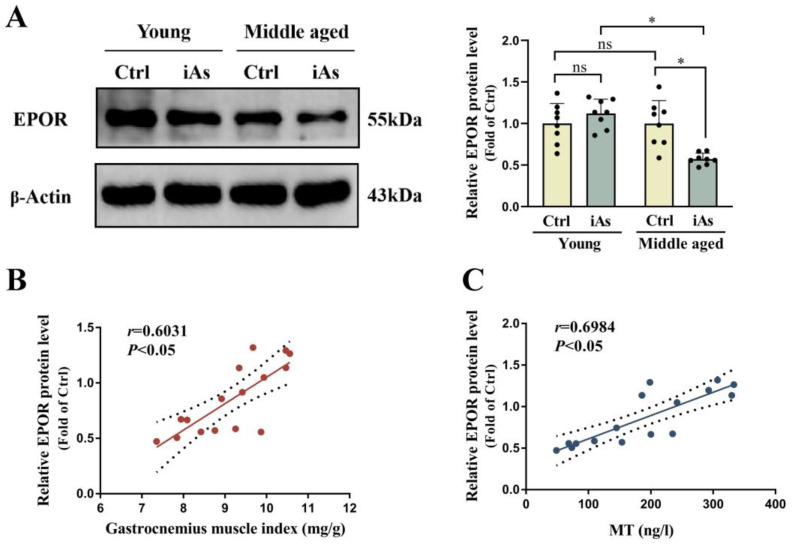
Impact of 1 mg/L arsenite exposure on EPOR expression of the gastrocnemius muscle (**A**) and its correlations with gastrocnemius muscle index and serum MT level (**B**,**C**) in 3- and 12-month-old rats. The relative EPOR protein level was positively correlated with gastrocnemius muscle index (*n* = 16, *r* = 0.6031, *p* < 0.05) and serum MT level (*n* = 16, *r* = 0.6984, *p* < 0.05). Ctrl, Control; iAs, Arsenite; MT, Melatonin; EPOR, erythropoietin receptor. The data are expressed as mean ±SD for eight rats per group. ns, no significance compared to the control group. * *p* < 0.05, significant difference compared to the control group.

The result of Western blot showed that EPOR protein expression level of the gastrocnemius muscle was significantly downregulated in 12-month-old arsenite-exposed rats while its expression had no statistically significant difference in 3-month-old arsenite-exposed rats (Figure 6A). To elucidate the possible regulatory effect of MT on EPOR and its role in arsenite-induced skeletal muscle injury, a correlation analysis among EPOR and MT as well as gastrocnemius muscle index was conducted. Correlation analysis showed that the EPOR protein level of the gastrocnemius muscle was positively correlated with serum MT and gastrocnemius muscle index (Figure 6B,C).

### 3.6. Effect of Exogenous MT on Arsenite-Induced Skeletal Muscle Injury in Middle-Aged Rats

**Figure 7:**
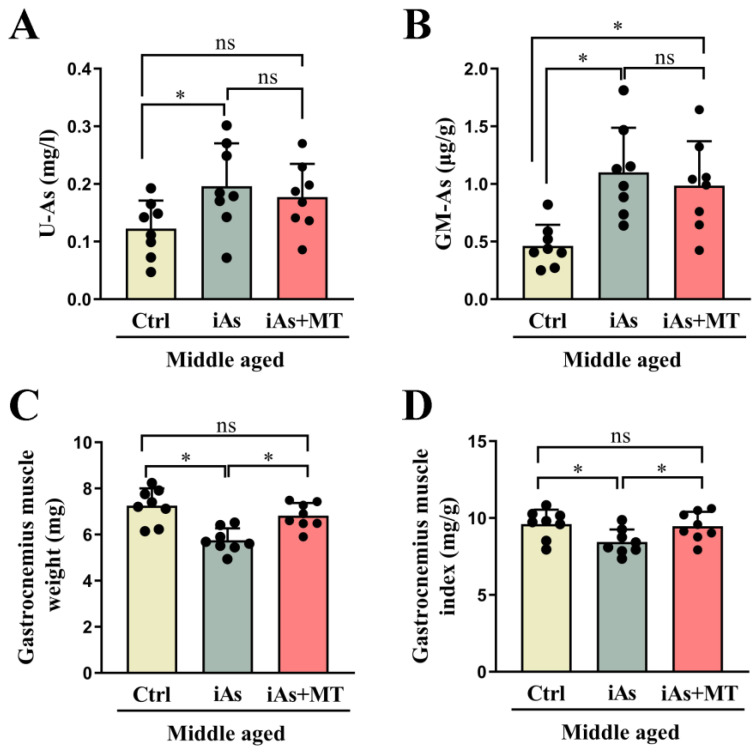
Effects of exogenous MT on arsenic concentrations in urine (**A**), gastrocnemius muscle (**B**), gastrocnemius muscle weight (**C**), and gastrocnemius muscle index (**D**) in 12-month-old rats. Ctrl, Control; iAs, Arsenite; U-As, Urine arsenic concentration; GM-As, Gastrocnemius muscle arsenic concentration. The data are expressed as mean ±SD for eight rats per group. ns, no significance compared to the control group. * *p* < 0.05, significant difference compared to the control group.

**Figure 8:**
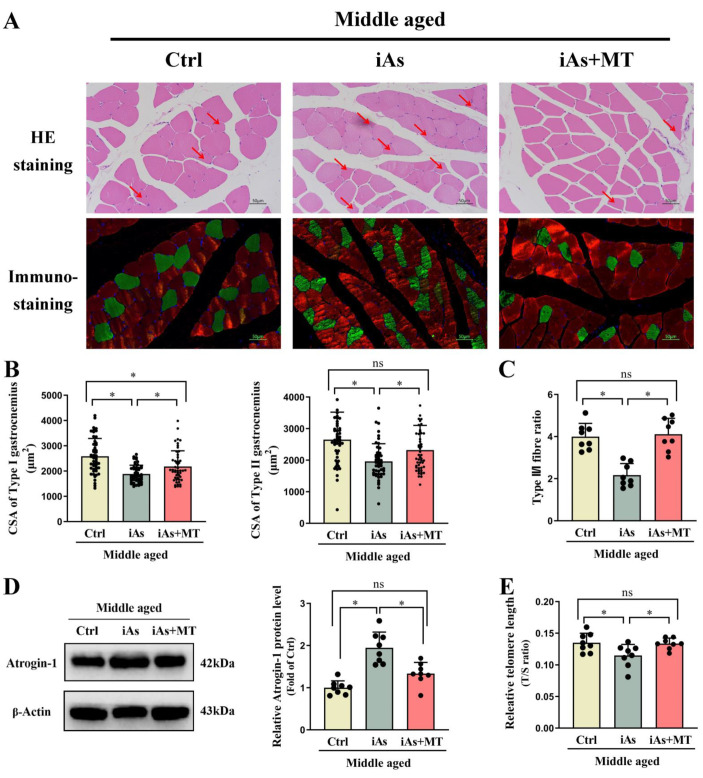
Effects of exogenous MT on pathological morphology (**A**), gastrocnemius muscle fiber cross-sectional area (**A**,**B**), myofiber type switching (**C**), the protein level of Atrogin-1 (**D**), and relative telomere length (**E**) of the gastrocnemius muscle in 12-month-old rats. Ctrl, Control; iAs, Arsenite. The data are expressed as mean ±SD for eight rats per group. Images (×200 magnification). Red arrows indicate atrophied skeletal muscle fibers. The green represents type I (slow-twitch) skeletal muscle fiber and the red represents type II (fast-twitch) skeletal muscle fiber. Scale bars represent 50 µm. * *p* < 0.05, significant difference compared to the control group.

**Figure 9:**
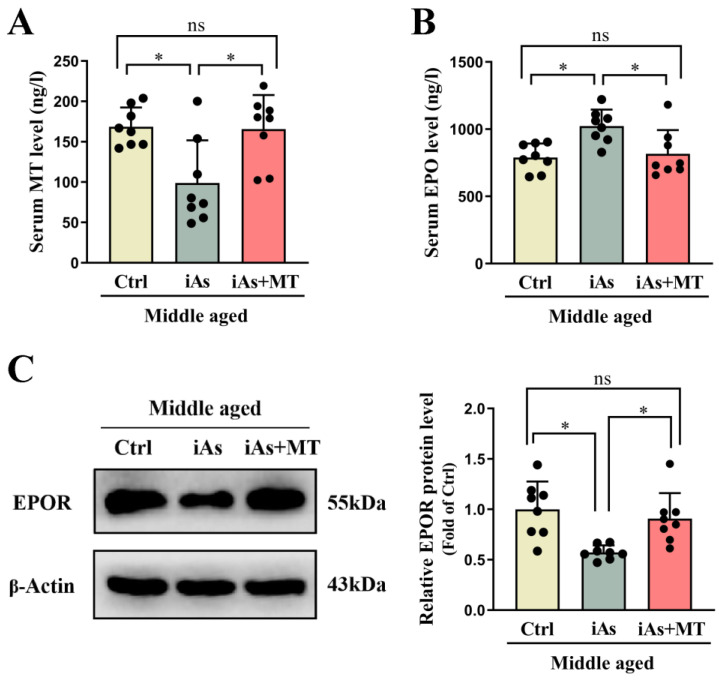
Effects of exogenous MT on the levels of serum MT and EPO (**A**,**B**), as well as EPOR expression of the gastrocnemius muscle (**C**) in 12-month-old rats. Ctrl, Control; iAs, Arsenite; MT, Melatonin; EPO, erythropoietin. The data are expressed as mean ±SD for eight rats per group. ns and *p* > 0.05, no significance compared to the control group. * *p* < 0.05, significant difference compared to the control group.

To further clarify the role of MT in arsenite-induced skeletal muscle injury, exogenous MT was used to investigate its effect on EPOR expression of skeletal muscle. The results showed that compared with the 12-month-old arsenite-exposure group, exogenous MT had no impact on arsenic concentration both in urine (Figure 7A) and gastrocnemius muscle (Figure 7B). Exogenous MT could effectively antagonize arsenite-induced decline of gastrocnemius muscle weight (Figure 7C) and gastrocnemius muscle index (Figure 7D), type I and type II gastrocnemius muscle atrophy, and myofiber type switching in 12-month-old rats (Figure 8A–C). Exogenous MT could also restore the protein level of Atrogin-1 and the relative telomere length of the gastrocnemius muscle in 12-month-old arsenite-exposed rats (Figure 8D,E). In addition, MT supplement recovered serum MT and EPO levels (Figure 9A,B), accompanied by EPOR expression of gastrocnemius muscle in 12-month-old arsenite-exposed rats (Figure 9C).

## 4. Discussion

Arsenic is a kind of environmental poison that can induce multiple organ damage. However, its impact on skeletal muscle and the underlying mechanisms are largely unknown. This study provided novel evidence around the influence of arsenite exposure on endogenous MT secretion and its effect on skeletal muscle EPOR protein expression and index while analyzing their correlations in an arsenite exposure rat model.

### 4.1. Arsenic Exposure and Skeletal Muscle Injury

The toxic effects of arsenic on skeletal muscle have been gradually recognized and are increasingly concerning. Recently, epidemiological studies have shown that long-term arsenic exposure is associated with reduced skeletal muscle mass loss [6,7]. A previous study has also found that exposure to 0.5 mg/L As_2_O_3_ could induce skeletal muscle atrophy in mice [8]. However, the pathological changes of skeletal muscle may also involve fatty degeneration, fibrosis, myofiber type switching, shortened telomeres, and so on. More importantly, skeletal muscle is characterized by an age-related decline from middle age in physiological conditions [36]. We selected young and middle-aged rats to investigate whether the skeletal muscle toxicity of arsenic had an age susceptibility. Among them, the 3-month-old rats represented the young rats, and the 12-month-old rats represented the middle-aged rats based on previous studies [37,38]. Therefore, we investigated the difference in the effects of sodium arsenite on pathological types of skeletal muscle of young and middle-aged rats. Interestingly, our results showed that 1 mg/L arsenite exposed for 3 months exhibited different toxic effects on gastrocnemius muscle between young and middle-aged rats. It could not only accelerate both type I and type II myofiber atrophy but also induce telomere shortening and type II to type I myofiber switching in gastrocnemius muscle of middle-aged rats, while it did not cause significant morphology changes to gastrocnemius muscle in young rats and did not induce significant steatosis and fibrosis in gastrocnemius muscle tissue. Furthermore, the amounts of arsenic in muscle were increased in 12-month-old rats, which might be derived from increased gastrointestinal absorption of arsenic or increased retention of arsenic in a body. These results indicated an increased susceptibility to skeletal muscle toxicity of arsenite in middle-aged rats.

### 4.2. MT in Arsenite-Induced Skeletal Muscle Injury

In general, skeletal muscle mass is affected by a variety of factors, such as neurotransmitters, hormones, inflammation, etc. Nevertheless, hormonal regulation is one of the most important regulatory factors of skeletal muscle mass [39]. A large number of studies have demonstrated that MT is an important hormone in maintaining skeletal muscle mass. Administration of exogenous MT can restore skeletal muscle mass in patients with sarcopenia [26,27]. The disturbance of MT secretion can lead to the loss of skeletal muscle mass [40]. In addition, more and more studies have shown that MT can effectively antagonize the systemic multi-organ and multi-system toxic effects of arsenic [41]. Based on these findings, we investigated the effects of arsenite on endogenous MT secretion in rats. We found that serum MT level increased in young arsenic exposed rats while decreased in middle-aged arsenic exposed rats. The former may be a stress response of arsenite-induced physical impairment, while the latter may be relevant to a decline in age-related responsiveness and compensatory function. Administration of exogenous MT significantly antagonized the toxic effect of arsenite on gastrocnemius muscle. These results further suggested that the disturbance of endogenous MT secretion may be a key factor in arsenite-induced skeletal muscle injury.

### 4.3. EPO Sensitivity in Arsenite-Induced Skeletal Muscle Injury

There is a growing body of evidence suggesting that EPO has a protective effect on skeletal muscle, and the increased secretion of EPO contributes to the repair of skeletal muscle injury [18,19,20]. In general, the expression of EPOR in organs or tissue cells is upregulated in response to increased peripheral blood EPO levels to increase organ or tissue sensitivity of EPO [21]. Recent studies have shown that arsenic can promote EPO secretion in rats and mice [23,24], which is consistent with our results. However, we found the change of EPOR expression in gastrocnemius muscle was opposite to the change in EPO levels in peripheral blood in middle-aged arsenite-exposed rats, suggesting that arsenite exposure could reduce the sensitivity of skeletal muscle to EPO in middle-aged rats.

**Figure 10:**
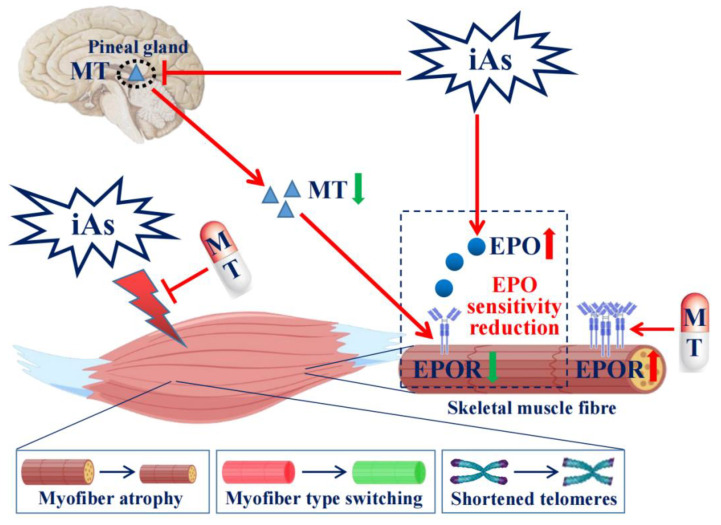
Proposed model of the role of MT secretion and its regulated EPO sensitivity of skeletal muscle in arsenite-induced skeletal muscle injury. Chronic environmental relevant concentration arsenite (iAs) exposure induced gastrocnemius muscle atrophy, type II to type I myofiber switching, and shortened telomeres, accompanied by declined serum MT level and EPOR protein expression of skeletal muscle as well as increased serum EPO level in middle-aged rats. Exogenous MT could effectively antagonize these toxic effects of arsenite.

In fact, the toxic effects of arsenite on skeletal muscle may be divided into direct toxicity and indirect toxicity. Numerous studies have shown that arsenite has cytotoxic effects. The results of this study showed that the accumulation of arsenic was increased in skeletal muscle of middle-aged rats, which may contribute to the EPO sensitivity decline in skeletal muscle. Importantly, studies have shown that MT can promote EPO sensitivity in anemic patients with chronic kidney disease or chronic renal failure [28,29]. Our results suggest that administration of exogenous MT could effectively restore EPOR expression in skeletal muscle and serum EPO level in middle-aged rats, indicating declined MT secretion may be an indirect factor contributing to arsenite-induced EPO sensitivity reduction of skeletal muscle. These results suggested that arsenite-induced EPO sensitivity decline in skeletal muscle may be due to arsenic’s skeletal muscle toxicity and suppressed MT secretion (Figure 10).

## 5. Conclusions

Taken together, this study reveals that arsenite exposure induced skeletal muscle atrophy and type II fiber reduction were associated with decreased MT secretion and EPO sensitivity to skeletal muscle. Exogenous MT supplementation effectively antagonized arsenite-induced skeletal muscle injury partially by increasing EPOR expression in skeletal muscle. This study could provide a new strategy for the prevention and treatment of arsenite-induced skeletal muscle injury in arsenic-contaminated areas.

## Data Availability

The data presented in this study are available upon request from the corresponding author.
